# Risks of SARS-CoV-2 on male reproductive health and the practice of semen analysis and cryopreservation

**DOI:** 10.2217/fmb-2020-0114

**Published:** 2020-11-06

**Authors:** Michael B Yakass, Osbourne Quaye, Bryan J Woodward

**Affiliations:** 1 West African Centre for Cell Biology of Infectious Pathogens (WACCBIP), University of Ghana, Accra, Ghana; 2 Department of Biochemistry, Cell & Molecular Biology, University of Ghana, Accra, Ghana; 3 Assisted Conception Unit, Lister Hospital & Fertility Centre, Accra, Ghana; 4 X & Y Fertility, Leicester, UK

**Keywords:** ACE2, COVID-19, fertility, male reproduction, SARS-CoV-2, semen

The COVID-19 pandemic has led to urgent action by fertility clinics across the world. Many countries took the decision to suspend fertility treatments to mitigate the risk of spreading the infection, following international professional body guidance (e.g., from the European Society of Human Reproduction and Embryology and from the American Society of Reproductive Medicine). Given the virulence of SARS-CoV-2 and our lack of knowledge of how it can affect medically assisted reproduction (MAR), suspension was the safest course of action.

Viruses are a concern for MAR. In 2005, the European Union issued directives to ensure that all patients are screened for three blood-borne viruses HIV, HBV and HCV, prior to embarking on any form of MAR. More recently, mosquito-borne viruses have also been shown to affect fertility [1]. For example, Zika virus RNA has been detected in semen of infected men for up to 1 year post-recovery, even after the patients are cleared of symptoms [2].

## Virus shedding into semen

Typically, viruses that present with very high titres in blood (viremia) have a high chance of being shed in other body fluids such as sweat, urine, feces, breast milk and semen. To date, some 27 viruses have been detected in human semen [3]. There is, therefore, a possibility that SARS-CoV-2 could shed into semen and potentially remain infective.

In addition to viremia, other factors that can influence the existence of viruses in semen include the immune response in the male reproductive tract, inflammatory mediators that may alter the blood-testis barrier and the structural stability of the virus [3]. The presence of certain viruses in semen may not necessarily be a function of the availability of cognate receptors for infection within the testes, or even the ability of the virus to replicate in male reproductive tissues. Considering the paucity of data on SARS-CoV-2, it is prudent to learn from other viruses that belong to the same family.

SARS-CoV-2 belongs to the family Coronaviridae, alongside SARS-CoV and MERS-CoV. These viruses cause similar pathogenesis in the upper and lower respiratory tracts. Infectious SARS-CoV was detected in blood, urine and stools, while infectious MERS-CoV was not detected in nonrespiratory fluid [4]. SARS-CoV-2 shares about 76% sequence similarity with SARS-CoV, and the two viruses infect host target cells by binding to the same cellular entry receptor: the angiotensin converting enzyme receptor 2 (ACE2) [5]. Initial bioinformatic analysis of single-cell RNA sequencing indicated abundant expression of ACE2 in type II alveolar cells [6]. Liver impairment was reported in about 60% of MERS-CoV and SARS-CoV infected patients [7,8] and liver damage has also been reported in COVID-19 patients [9], possibly via infection of cholangiocytes in the liver.

Diarrhea has been shown as one of the symptoms in COVID-19 patients, and the putative receptor ACE2 expression has been identified in absorptive enterocytes in the ileum and colon by using single-cell RNA sequencing; suggesting a potential route of infection of SARS-CoV-2 in the GI tract [10]. Single-cell RNA sequencing analysis also reveals multiple human physiologic organs and systems that may be targets for SARS-CoV-2 infection including myocardial cells, proximal tubules of the kidney, bladder and urothelial cells [11].

SARS-CoV-2 viral RNA has been detected in blood and feces, but recovery of viable viruses is yet to be observed [12,13]. It is worth noting that in stool specimens, very low titres of SARS-CoV-2 were detected by quantitative PCR (RT-qPCR) with high cycle threshold/Cq values in the range 36–38 [13]. Based on the low titres detected from stools, it seems that the risk of significant SARS-CoV-2 shedding in human semen may be low. Furthermore, reports of low expression of the ACE2 receptor in male gamete and testicular cells have also been made [14]. However, single-cell RNA sequencing analysis shows predominant expression of the ACE2 receptor in spermatogonia, Leydig and Sertoli cells [15], suggesting that human reproductive organs could be possible targets of SARS-CoV-2 replication.

At the time of writing, two studies have reported no detection of SARS-CoV-2 viral RNA by RT-qPCR in semen or testicular tissue; one study from ten COVID-19 deceased patients [16] and another study from 34 males recovering from mild symptoms of COVID-19 [14]. However, the authors agree that their results were impacted by the small sample size and selection bias, as the men with COVID-19 in these two studies showed only mild symptoms and may not have had high viremia. These groups stated that they could not definitively rule out the presence of SARS-CoV-2 in the seminal fluid during an acute infection with severe COVID-19 symptoms.

One study has now reported that SARS-CoV-2 has been detected in the semen of four out of 15 acutely ill and two out of 23 men recovering from COVID-19 [17]. This raises concerns about the possibility of sexual transmission of SARS-CoV-2. However, it must be stressed that this is a single study with a small sample size and it is unknown if the detected virus is infectious in semen. Further larger studies are needed to give credence or refute the idea of SARS-CoV-2 detection in semen of acutely infected men. Furthermore, to date there is no evidence that SARS-CoV-2 can remain infectious in the semen months after infection, as has been reported in the case of Zika virus [2].

The absence of infectious virus from semen does not preclude the possibility of SARS-CoV-2 affecting the male reproductive tract. SARS-CoV caused orchitis with widespread destruction of germ cells, such that the number of spermatozoa was severely reduced in the seminiferous tubules of six men who died from the disease [18]. However, it should be stressed that SARS-CoV viral RNA was not detected in the testes by *in situ* hybridization. Moreover, Xu and colleagues reported higher levels of lymphocytes and macrophages in testicular interstitial tissue and suggested that the observed SARS-induced orchitis may not be due to viral replication but the inflammatory response in testicular tissues [18].

About 20% of 34 males recovering from mild symptoms of COVID-19 have reported experiencing scrotal discomfort suggestive of orchitis, but the authors could not investigate this further [14]. Taking into account the holistic and prognostic wellbeing of SARS-CoV-2 infected men, it may prove beneficial for healthcare specialists to probe and investigate the possibility of SARS-CoV-2 induced orchitis. Perhaps with early detection and appropriate remedial action, the fertility of SARS-CoV-2 infected men can be preserved should there be the case of similar disease sequelae as may have been observed with SARS-CoV previously.

Weighing up the evidence, and considering the presence of the blood–testes barrier, we suggest that there will be a negligible chance to have SARS-CoV-2 in semen of mildly infected COVID-19 patients, as already demonstrated in the report from Wuhan [14]. The real risk of detecting SARS-CoV-2 in semen will be in acute and severely infected patients with high SARS-CoV-2 viremia. However, the likelihood of such infected men wanting to have sexual intercourse is minimal. This also brings a practical and ethical challenge of collecting semen from severely ill COVID-19 patients, which may hinder the quest for larger studies to find stronger empirical evidence for the presence of SARS-CoV-2 in semen and the associated impact that could have on the male reproductive tract.

### Risk of SARS-CoV-2 transmission to laboratory personnel during semen analysis

Although many fertility clinics and pathology labs suspended semen analysis during the height of the SARS-CoV-2 pandemic, other labs remained open. Even where diagnostic semen analysis was suspended, many pathology labs continued to perform postvasectomy semen analyses, indicating that they considered the risk of transmission to be minimal [19].

Provided best laboratory practice is adhered to, then utmost caution should be taken when performing any type of semen analysis or semen processing for fertility treatment. Given that SARS-CoV-2 is an air-borne virus, additional care should be undertaken to avoid exposure of aerosols. Aerosol-generating events include the opening of specimen containers used to collect semen and the tubes used in sample centrifugation. During processing all tube lids should be tightly fitted and centrifuges should be used with aerosol-tight caps of centrifuge buckets. While staff in the laboratories of fertility clinics may wear appropriate personal protective equipment (PPE), these steps should become part of standard practice, if not already in use.

## Survival of viruses in liquid nitrogen?

Taking a cue from standard practice of cryopreserving viruses in laboratories, most viruses remain viable almost indefinitely at ultra-low temperatures if stored dried, in appropriate protein concentrations and between pH of 7 and 8 [20]. For example, the influenza virus (an enveloped RNA virus belonging to the Orthomyxoviridae family) causes similar respiratory tract pathogenesis to SARS-CoV-2 and has been reported to survive and maintain viability when stored in the vapor phase of liquid nitrogen for up to 40 years [21]. SARS-CoV-2 is also an enveloped RNA virus and, although there is no empirical data, it is theoretically possible that the virus could remain viable when thawed after cryopreservation in liquid nitrogen.

To date, there is no evidence in the field of MAR for cross-contamination of any virus from one clinical sample to another while stored in liquid nitrogen or vapor phase storage. If SARS-CoV-2 was shed into a semen sample and cryopreserved in liquid nitrogen, there is almost no chance of cross-contaminating other samples while cryopreserved. However, best practice should be adhered to in MAR cryobanks, with storage of all samples in hermetically-sealed high security devices, with quarantined samples segregated from fully screened samples.

## Conclusion

At the time of writing, there are contradictory reports as to whether SARS-CoV-2 can be shed into semen of infected men. The current evidence could be considered poor quality due to the low patient numbers. As a precaution, men recovering from COVID-19 may wish to undergo a semen analysis to confirm if their fertility status has been affected by the illness.

On a constructive note, the pandemic has provided an opportunity for fertility clinics to review their current practices and share perspectives with other professionals. As such, dissemination of good laboratory practice in fertility clinics worldwide may be a silver lining in the cloud cast by the COVID-19 pandemic.
